# Chemogenomic Screening in a Patient‐Derived 3D Fatty Liver Disease Model Reveals the CHRM1‐TRPM8 Axis as a Novel Module for Targeted Intervention

**DOI:** 10.1002/advs.202407572

**Published:** 2024-11-28

**Authors:** Sonia Youhanna, Aurino M. Kemas, Shane C. Wright, Yi Zhong, Britta Klumpp, Kathrin Klein, Aikaterini Motso, Maurice Michel, Nicole Ziegler, Mingmei Shang, Pierre Sabatier, Aimo Kannt, Hongda Sheng, Nuria Oliva‐Vilarnau, Florian A. Büttner, Brinton Seashore‐Ludlow, Jonas Schreiner, Maike Windbergs, Martin Cornillet, Niklas K. Björkström, Andreas J. Hülsmeier, Thorsten Hornemann, Jesper V. Olsen, Yi Wang, Roberto Gramignoli, Michael Sundström, Volker M. Lauschke

**Affiliations:** ^1^ HepaPredict AB Stockholm 17165 Sweden; ^2^ Department of Physiology and Pharmacology Karolinska Institutet Stockholm 17165 Sweden; ^3^ Center for Molecular Medicine Karolinska Institutet and University Hospital Stockholm 17176 Sweden; ^4^ Pharmaceutical Informatics Institute College of Pharmaceutical Sciences Zhejiang University Hangzhou Zhejiang 310058 China; ^5^ Dr. Margarete Fischer‐Bosch Institute of Clinical Pharmacology (IKP) 70376 Stuttgart Germany; ^6^ University of Tübingen 72074 Tübingen Germany; ^7^ Department of Oncology and Pathology Science for Life Laboratory Karolinska Institutet Stockholm 17164 Sweden; ^8^ Fraunhofer Institute for Translational Medicine and Pharmacology (ITMP) 60596 Frankfurt am Main Germany; ^9^ Division of Rheumatology Department of Medicine Karolinska University Hospital and Karolinska Institutet Stockholm 17164 Sweden; ^10^ Novo Nordisk Foundation Center for Protein Research Faculty of Health and Medical Sciences University of Copenhagen Copenhagen 2200 Denmark; ^11^ Cardio‐Thoracic Translational Medicine Lab Department of Surgical Sciences Uppsala University Uppsala 75185 Sweden; ^12^ Institute of Clinical Pharmacology Goethe University 60596 Frankfurt Germany; ^13^ Institute of Pharmaceutical Technology Goethe University Frankfurt 60438 Frankfurt am Main Germany; ^14^ Centre for Infectious Medicine Department of Medicine Huddinge Karolinska University Hospital Karolinska Institutet Huddinge 14152 Sweden; ^15^ University of Zurich University Hospital Zurich Institute of Clinical Chemistry Zurich 8091 Switzerland; ^16^ State Key Laboratory of Component‐based Chinese Medicine Tianjin University of Traditional Chinese Medicine Tianjin 301617 China; ^17^ Department of Laboratory Medicine Division of Pathology Karolinska Institutet Stockholm 17177 Sweden; ^18^ Department of Pathology and Cancer Diagnostics Karolinska University Hospital Huddinge 17164 Sweden; ^19^ Department of Pharmacy the Second Xiangya Hospital Central South University Changsha 410011 China

**Keywords:** chemical probes, fibrosis, muscarinic receptor, NASH, phenotypic assay, target discovery

## Abstract

Metabolic dysfunction‐associated steatohepatitis (MASH) is a leading cause of chronic liver disease with few therapeutic options. To narrow the translational gap in the development of pharmacological MASH treatments, a 3D liver model from primary human hepatocytes and non‐parenchymal cells derived from patients with histologically confirmed MASH was established. The model closely mirrors disease‐relevant endpoints, such as steatosis, inflammation and fibrosis, and multi‐omics analyses show excellent alignment with biopsy data from 306 MASH patients and 77 controls. By combining high‐content imaging with scalable biochemical assays and chemogenomic screening, multiple novel targets with anti‐steatotic, anti‐inflammatory, and anti‐fibrotic effects are identified. Among these, activation of the muscarinic M_1_ receptor (CHRM1) and inhibition of the TRPM8 cation channel result in strong anti‐fibrotic effects, which are confirmed using orthogonal genetic assays. Strikingly, using biosensors based on bioluminescence resonance energy transfer, a functional interaction along a novel MASH signaling axis in which CHRM1 inhibits TRPM8 via G_q/11_ and phospholipase C‐mediated depletion of phosphatidylinositol 4,5‐bisphosphate can be demonstrated. Combined, this study presents the first patient‐derived 3D MASH model, identifies a novel signaling module with anti‐fibrotic effects, and highlights the potential of organotypic culture systems for phenotype‐based chemogenomic drug target identification at scale.

## Graphical abstract

A novel patient‐derived 3D MASH model is presented that closely mimics key disease hallmarks at the molecular and functional level and offers a pathophysiologically relevant platform for high‐throughput drug discovery. Phenotypic and chemogenomic screening using this model uncover novel druggable targets. This platform promises to improve the understanding of disease mechanisms and facilitate the accelerated discovery of effective new drugs.

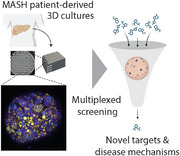

## 1 Introduction

Metabolic dysfunction‐associated steatotic liver disease (MASLD), until recently termed nonalcoholic fatty liver disease, constitutes a spectrum of liver disease that is characterized by excess levels of intracellular triglycerides in ≥5% of hepatocytes. MASLD ranges from initially benign steatosis to metabolic dysfunction‐associated steatohepatitis (MASH), fibrosis, and cirrhosis. With a global prevalence of 32% in the general adult population and up to 90% in morbidly obese individuals, MASLD constitutes the leading cause of liver‐related morbidity and mortality^[^ ^1^, ^2^ ^]^ and has surpassed hepatitis C virus infection as the most common indication for liver transplantation in women^[^ ^3^ ^]^ and the elderly.^[^ ^4^ ^]^

Despite this high disease burden and a multitude of suggested targets, there is a lack of effective pharmacological treatments that directly target MASLD. While the first drug for MASH has recently been approved by the US Food and Drug Administration (FDA), Madrigal Pharmaceuticals’ resmetirom, the vast majority of candidates has failed in clinical trial. Notable recent cases include selonsertib, elafibranor, firsocostat, and cilofexor, which were all terminated due to lack of efficacy, as well as obeticholic acid, which failed due to safety concerns.^[^ ^5^ ^]^ Reasons for these failures are complex and multifactorial; however, the large translational gap resulting from discordance between preclinical models and clinical outcomes, and insufficient understanding of the molecular mechanisms underpinning the disease have been consistently identified as the central features.^[^ ^6^ ^]^ While animal models have played important roles in the preclinical evaluation of many compounds for efficacy and safety, they are increasingly recognized as being poor predictors, particularly in inflammatory and metabolic diseases.^[^ ^7^, ^8^, ^9^, ^10^ ^]^ This is evident in MASH, where none of the available dietary and genetic models fully reflect human disease etiology and progression or accurately mirror the underlying fibrogenic mechanisms.^[^ ^11^ ^]^

Human in vitro models aim to recapitulate key molecular aspects of MASH pathogenesis and to allow controlled investigations into the underlying mechanisms as well as rapid and scalable testing of candidate molecules.^[^ ^12^ ^]^ Most in vitro MASH models have used monolayer cultures of hepatic cell lines due to their better availability and lower costs. However, the molecular configuration and functionality of these cells are clearly distinct from hepatic in vivo phenotypes, which limits their utility for translational applications.^[^ ^13^ ^]^ Organotypic and microphysiological culture methods of primary cells promise to overcome these limitations and are increasingly adopted in drug discovery and development.^[^ ^14^, ^15^, ^16^ ^]^ These trends are further accelerated by the FDA Modernization Act, which removed the blanket mandate for animal testing for newly developed drugs, thus opening possibilities for replacement with physiologically or pathophysiologically relevant human tissue models.^[^ ^17^ ^]^

For the co‐culture of primary human hepatocytes (PHH) and non‐parenchymal cells (NPCs), different 3D culture systems have been developed that allow the maintenance of the mature functionality of cultured cells.^[^ ^18^, ^19^, ^20^, ^21^ ^]^ Specifically human liver spheroids are increasingly utilized for a multitude of applications within translational pharmacology.^[^ ^22^ ^]^ Liver spheroids preserve molecular phenotypes at the transcriptomic, proteomic, and metabolomic levels for multiple weeks.^[^ ^23^, ^24^, ^25^ ^]^ They furthermore accurately mimic steatosis, insulin resistance, and fibrosis, and have been shown to recapitulate the clinical effects of MASH‐candidate drugs.^[^ ^26^, ^27^, ^28^, ^29^, ^30^, ^31^ ^]^ However, in all these models, MASH needed to be induced in vitro and no model derived from patient cells has so far been presented.

Here, we present a scalable and long‐term stable 3D culture model based on liver cells from patients with histologically confirmed MASH. These patient‐derived spheroids exhibit increased steatosis with hepatocyte ballooning, increased levels of pro‐inflammatory cytokines, and elevated collagen deposition indicative of hepatic scarring. Comparison of molecular spheroid signatures with biopsy data from 306 MASH patients furthermore showed excellent alignment and accurate recapitulation of the different disease stages (metabolically healthy, steatosis‐only, and MASH). Using transcriptomic, proteomic, phosphoproteomic, and lipidomic approaches, we demonstrate significant alterations in extracellular matrix (ECM) organization, cholesterol biosynthesis, and translation, driven at least in part by altered activity of casein kinase. We benchmarked model performance by screening drug candidates currently in clinical trials, showcased its use in a drug discovery campaign targeting DNA damage and show compatibility of the system with nanoparticle‐based compound delivery. Last, we demonstrate the suitability of the system as a phenotypic platform for chemogenomic screening. The results pinpoint activation of the M_1_ muscarinic receptor (CHRM1) and inhibition of the TRPM8 ion channel as a novel disease module, which could be confirmed by orthogonal genetic approaches and in‐depth profiling of molecular interactions using bioluminescence resonance energy transfer (BRET).

## 2 Results

### 2.1 A Patient‐Derived 3D Cell Culture Model of Human MASH

To generate 3D spheroid cultures, PHH and NPCs were aggregated in ultra‐low attachment plates (ULA) over the course of 7 days. Primary human liver NPCs contained T‐cells (CD3^+^) with a smaller number of macrophages (CD14^+^), NK cells (CD56^+^), and B‐cells (CD19^+^ HLA‐DR^+^; Figure S1, Supporting Information). As expected, T cells were mainly CD4^+^ CD8^−^ in peripheral blood mononuclear cells (PBMCs) but CD4^−^ CD8^+^ in hepatic NPCs, recapitulating observations in the human liver. Among T cells, 5–20% were mucosal‐associated invariant T cells (MAIT cells; CD161^+^ TCRVα7.2^+^). As expected, we observed an increased proportion of MAIT cells in NPCs as compared to PBMCs. For CD56^+^ NK cells, 50–80% were CD16^−^, suggesting tissue residency.^[^ ^32^ ^]^ Compared to PBMCs, the increased proportion of CD16^−^ NK cells as well as their predominantly immature profile, characterized by an increased proportion of the NKG2A^+^ KIR^−^ subset,^[^ ^32^ ^]^ was also typical of the human intrahepatic immune system. Macrophages differed between donors in their polarization. While some donors contained >80% M1 macrophages (CD163^−^ CD206^−^), another donor contained almost 50% of M2 macrophages (CD163^+^, CD206^+^ or both). Overall, these data demonstrate that the immune cell repertoire of liver NPC isolates recapitulates the hallmarks of the human intrahepatic immune system. However, their integration and stability in culture remain to be determined.

**Figure 1:**
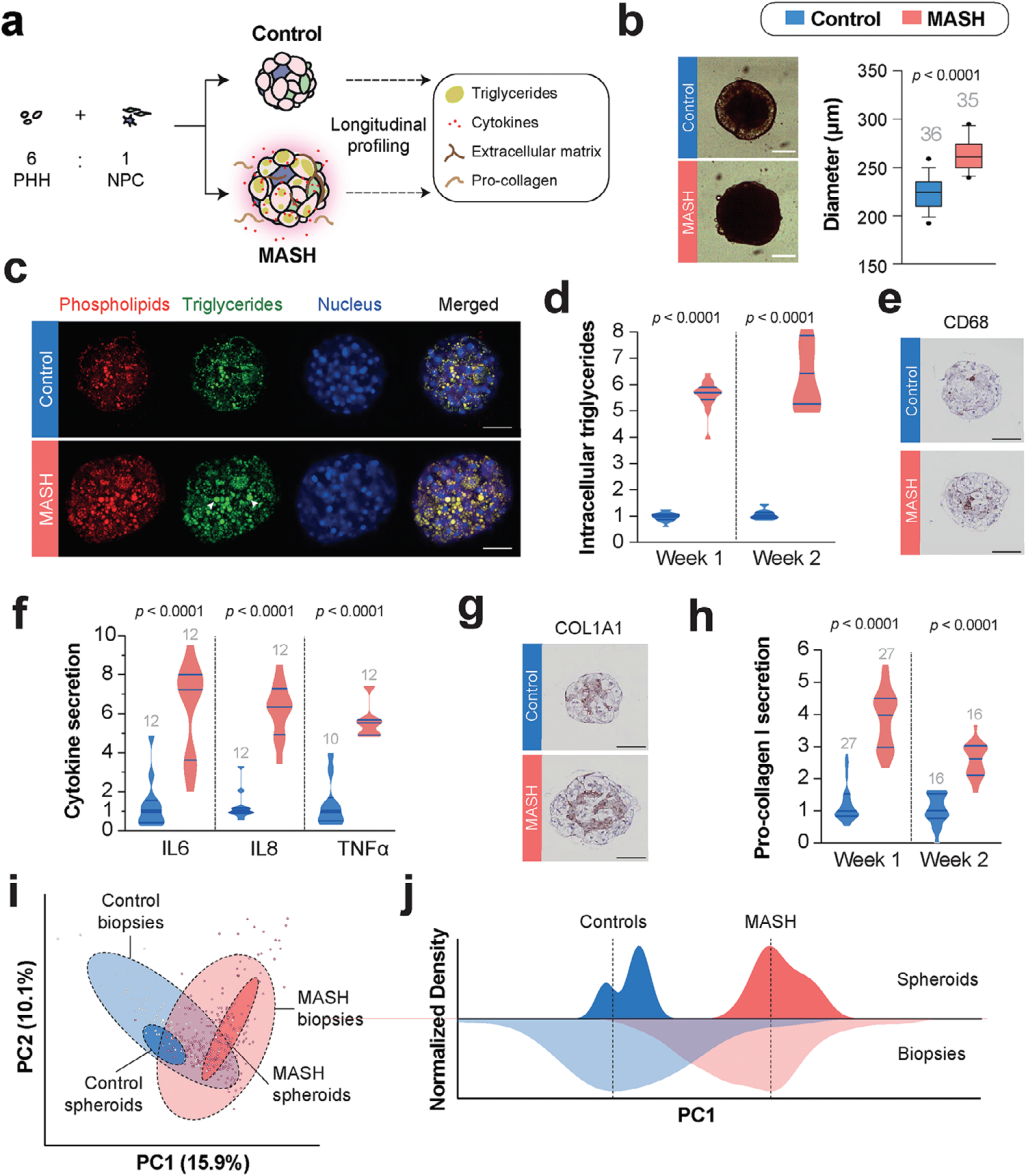
Organotypic primary human MASH cultures recapitulate key disease endpoints. a) Schematic depiction of the model setup. Primary human hepatocytes (PHH) and non‐parenchymal cells (NPC) are co‐cultured and molecular phenotypes of MASH and controls, including triglyceride levels, cytokines, pro‐collagen secretion, and collagen deposition, are compared over time. b) MASH cultures are significantly hypertrophic after two weeks in culture. Small grey numbers indicate the number of spheroids analyzed. c) Nile Red whole‐mount staining shows excessive levels of both phospholipids and intracellular triglycerides, as well as evidence of hepatocyte ballooning in MASH cultures (arrowheads). d) Biochemical quantifications reveal highly significant elevations of triglycerides in MASH cultures. Data is shown as fold change compared to control. e) Immunohistochemical staining against CD68. Note the increased number of CD68+ cells in MASH cultures. f) Secretion of pro‐inflammatory cytokines was significantly increased in MASH cultures. Data is shown as fold change compared to control. Small grey numbers indicate the number of wells (each containing ≈80 spheroids) across 2 independent seedings. g) Immunohistochemistry against COL1A1 reveals increased collagen deposition. h) Secretion of pro‐collagen I was significantly increased in MASH cultures. Data is shown as fold change compared to control. Small grey numbers indicate the number of wells (each containing ≈80 spheroids) across 3 independent seedings. i) Principal component analysis (PCA) of differential gene expression patterns based on RNA‐Seq analyses of spheroid data (dark red and blue ovals) and MASH patient biopsy samples from six cohort studies (light red and blue ovals; see Experimental Section). j) Density distribution of MASH and control samples along principal component 1 (PC1). Note that the densities of control and MASH are in very good agreement between spheroid cultures and patient biopsies. Scale bars = 100 µm. Error bars represent SEM.

Upon aggregation, spheroids were cultured in a medium containing pathophysiological concentrations of free fatty acids (**Figure** **1**a). Over the course of two weeks, accumulation of intracellular triglycerides, secretion of pro‐inflammatory cytokines as well as ECM remodeling were monitored. MASH microtissues exhibited significant hypertrophy compared to control spheroids (263 ± 15 µm SEM in MASH compared to 224 ± 15 µm SEM in controls; *p* < 0.0001; Figure 1b). Nile red staining revealed an increased number of lipid droplets in MASH microtissues, which aligned with the 5.6‐fold elevated intracellular triglyceride levels determined by biochemical quantification (Figure 1c,d). Immunohistochemical (IHC) staining against the monocyte marker CD68 confirmed the presence of liver macrophages (Figure 1e). In MASH samples, we observed a significantly higher secretion of IL6, IL8, and TNFα, suggesting an increasingly pro‐inflammatory milieu in line with MASH pathophysiology (Figure 1f). Importantly, MASH cultures also displayed increased deposition of COL1A1 and elevated secretion of pro‐collagen Ia1 (Figure 1g,h).

To contextualize the observed changes with alterations in MASH in vivo, we performed RNA‐sequencing of MASH and control spheroids and compared the transcriptomic signatures with cohort data from six sequencing studies of liver biopsies comprising a total of 306 MASH patients and 77 controls (see Experimental Section). To this end, we obtained the raw cohort sequencing data from GEO and harmonized analyses by applying the same analytical pipeline to both spheroid and biopsy data. We observed a clear separation of MASH and controls in both biopsy and spheroid samples (Figure 1i). Importantly, MASH spheroid samples overlapped with the MASH biopsy group whereas the control spheroids were clearly distinct and aligned with the control cohorts. Group separation was particularly pronounced along principal component 1 (PC1), and the density distribution of spheroid and biopsy samples clearly overlapped along this axis (Figure 1j). Combined, these results demonstrate that the developed patient‐derived 3D model recapitulates key hallmarks of MASH, including steatosis, inflammation, and fibrosis, and closely recapitulates in vivo disease signatures at the systems level.

### 2.2 Liver Spheroids Phenocopy MASH Hallmarks at the Transcriptomic, Proteomic, and Lipidomic Level

**Figure 2:**
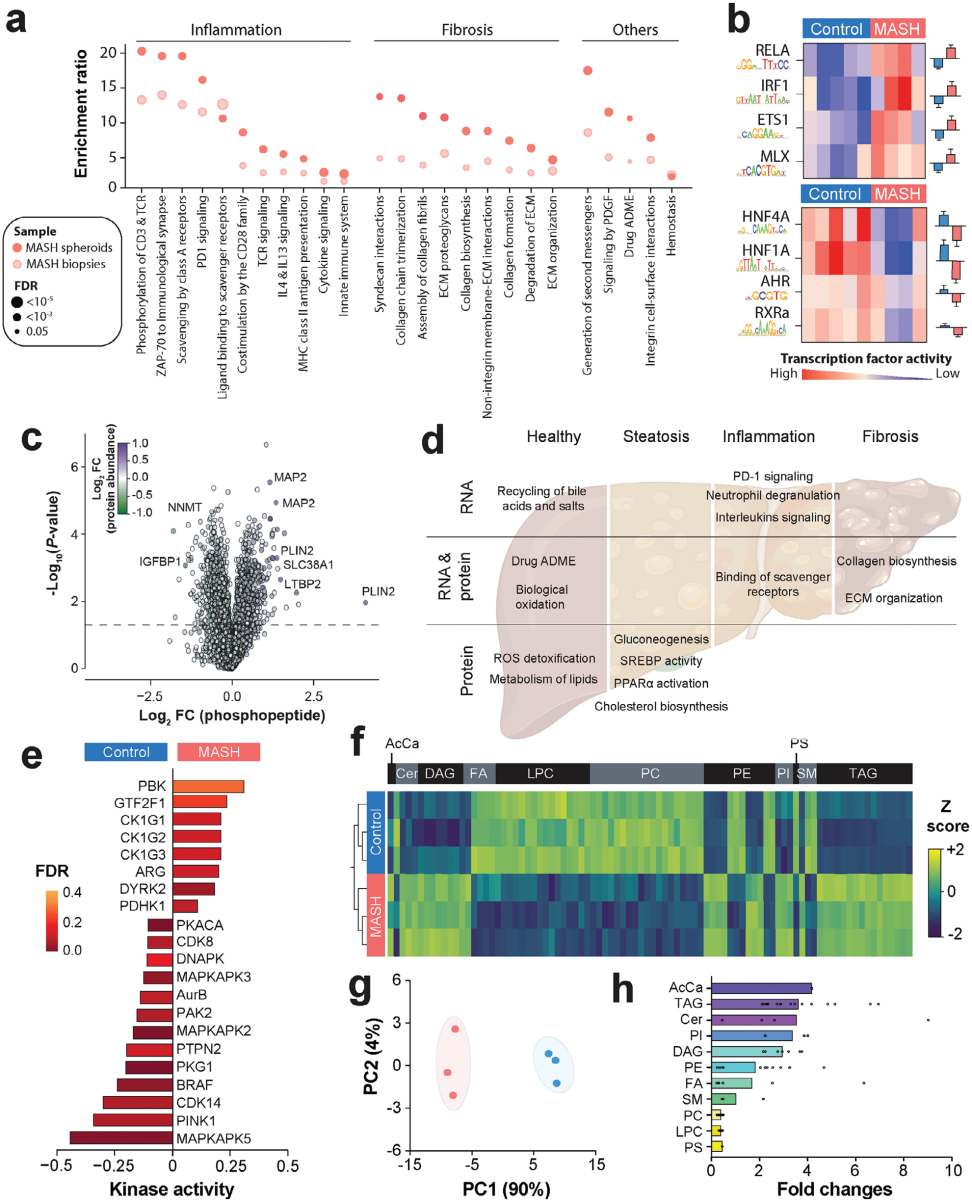
In‐depth profiling of the 3D patient‐derived MASH model using a multi‐omics approach. a) Reactome analysis of differentially regulated pathways based on transcriptomic data in MASH spheroids and MASH liver biopsies. Light red and dark red dots show enrichment in MASH biopsies and MASH spheroids, respectively. The dot sizes represent the false discovery rate (FDR). b) To understand the responsible transcription factors, we used enhancer motif inference based on available chromatin binding data using ISMARA. The most significantly altered transcription factors are shown with their corresponding binding motifs. c) Volcano plot showing significantly altered phosphopeptides in MASH versus controls. The *x‐*axis indicates the log2 fold change (FC) of phosphopeptide abundance while the color code indicates alterations of overall protein abundance. *n* = 3 biological replicates per group. d) Schematic depiction summarizing differentially regulated pathways associated with steatosis, inflammation, or fibrosis in MASH spheroids. Pathways that were only identified in transcriptomic or proteomic data are shown in the upper and lower third, respectively, while pathways that were found in both data sets are shown in the middle third. See Tables S3 and S5 (Supporting Information) for the detailed list of differentially regulated pathways in both data sets. e) Kinase activity inference analysis of phosphopeptide FC between MASH and control. Each bar represents the relative activity of the respective enriched kinase. The color represents the FDR of the enrichment. f) Heatmap visualization of differences in lipid composition between control and MASH spheroids across different lipid classes. Only lipids that differed greater than twofold and with *q *< 0.05 between the groups are shown. g) Principal component analysis (PCA) of lipidomic signatures indicates that differences between MASH and control spheroids (PC1; 90%) outweigh intra‐group variabilities (PC2; 4%). h) Bar plot of fold changes between MASH and control by lipid classes. Each differentially abundant lipid species within the class is indicated by separate dots. AcCa = acetylcarnitine; Cer = ceramides; DAG = diacylglycerols; FA = fatty acids; LPC = lysophosphatidylcholines; PC = phosphatidylcholines; PE = phosphatidylethanolamine; PI = phosphatidylinositol; PS = phosphatidylserine; SM = sphingomyelin; TAG = triacylglycerols.

Next, we conducted multi‐omics profiling of MASH spheroids. Our RNA‐sequencing analysis revealed significant differences in transcriptomic signatures. Pathway analyses showed that differentially expressed genes were enriched in factors involved in ECM organization, collagen deposition, and a multitude of immune functions, such as PD‐1 and interleukin signaling (**Figure** **2**a, Table S3, Supporting Information). Importantly, these results in spheroids showed excellent alignment with transcriptomic changes at the pathway level observed in MASH liver biopsies. This lends further support to the accurate mirroring of in vivo pathophysiological changes in patient‐derived MASH spheroids.

Global analysis of the activities of 500 transcription factors using promoter motif inference (see Experimental Section) showed that activities of RELA and IRF1, known mediators of steatosis, liver inflammation, and injury,^[^ ^33^, ^34^, ^35^ ^]^ were significantly increased in MASH (Figure 2b, Table S4, Supporting Information). Similarly, MLX, a key regulator of hepatic lipid and carbohydrate metabolism,^[^ ^36^ ^]^ and ETS1, a transcription factor involved in fibrosis development,^[^ ^37^ ^]^ showed increased activity in MASH. In contrast, the activity of HNF4α and HNF1α, which are central regulators of hepatic differentiation and known to inhibit epithelial‐to‐mesenchymal transition in liver fibrosis,^[^ ^38^, ^39^ ^]^ were significantly reduced. Activities of RXRa and AHR were also decreased; RXRa represses liver fibrosis by blocking hepatic stellate cell activation and proliferation,^[^ ^40^ ^]^ while AHR modulates inflammasome activation through inhibition of NF‐kB.^[^ ^41^ ^]^

To garner insights at the functional level, we complemented RNA‐sequencing with mass spectrometry‐based proteomic and phosphoproteomic assessments (Figure 2c). PLIN2, a known mediator of hepatic steatosis, inflammation, and fibrosis in mice,^[^ ^42^, ^43^ ^]^ was increased in both total protein and phosphorylation. Similarly, we observed a surge in phosphorylation of proteins involved in ECM rearrangement and tissue remodeling, such as MAP2 and LTBP2, as well as increased levels of SNAT1 (*SLC38A1*), a glutamine transporter implicated in stellate cell activation.^[^ ^44^ ^]^ Pathway analyses of proteomic data provided orthogonal support of the observed changes at the transcriptomic level (Table S5, Supporting Information). Specifically, activation of pathways involved in inflammation, collagen formation, and ECM remodeling was identified at both transcript and protein levels. In contrast, metabolic alterations, such as increased cholesterol biosynthesis and reduced IGF transport were predominantly identified in proteomic data (Figure 2d). Phosphorylation site motif analyses indicated PBK, kinases of the casein kinase 1γ family, and DYRK2 as putative upstream regulators (Figure 2e).

Lipidomic analysis showed that 87 out of 265 lipids were differentially abundant between the groups (greater than or equal to twofold change and *q *< 0.05; Table S6, Supporting Information). Levels of acyl carnitin (AcCa), diacyl‐ and triacyl‐glycerides (DAG and TAG, respectively), and most ceramides were increased, whereas phosphatidylserine (PS), lysophosphatidylcholines (LPC) and phosphatidylcholines (PC) were depleted in MASH (Figure 2f–h). Notably, fatty acid content remained overall unchanged between MASH and controls, in agreement with previous reports.^[^ ^45^ ^]^

### 2.3 Benchmarking of Pharmacological Modulation Using MASH Drug Candidates

**Figure 3:**
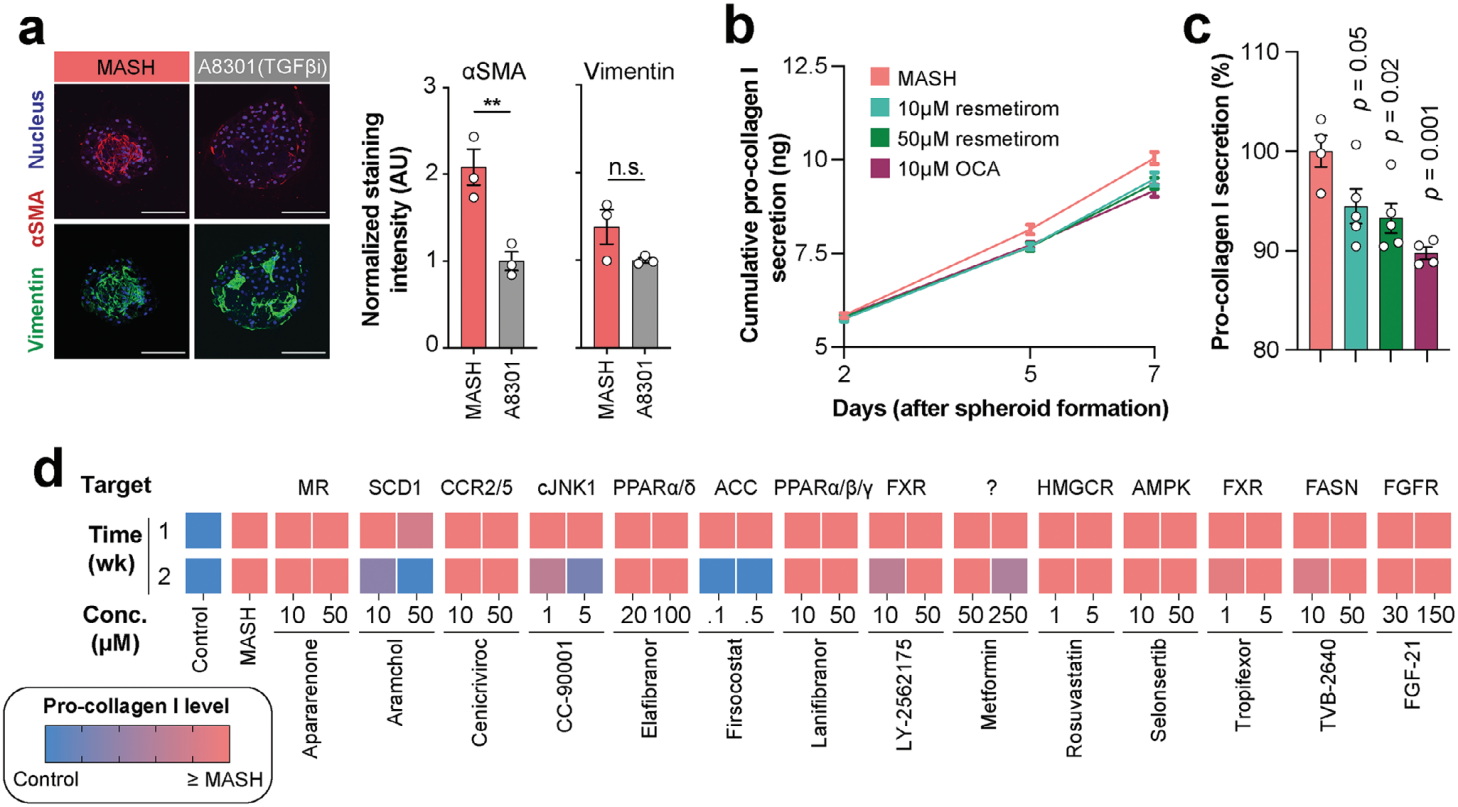
The patient‐derived MASH model is amenable to pharmacological interventions. a) Treatment of MASH microtissues for two weeks with the TGFβ receptor inhibitor A8301 significantly curtailed stellate cell activation (αSMA) without affecting stellate cell numbers (vimentin). For quantification, αSMA and vimentin signals were normalized to the nucleus number. *n* = 3 per group. Scale bar = 100 µm. b) Resmetirom and obeticholic acid (OCA) treatment alleviates fibrosis in MASH microtissues. Pro‐collagen I levels were measured in cell culture supernatants after different times of treatment, indicating that effects are detectable as early as 5 days after exposure. *n* = 4 per group and time point. c) The relative reduction in pro‐collagen levels after 7 days is significant for both treatment groups. d) MASH spheroids were exposed to 14 clinical anti‐MASH candidates representing a diverse range of targets and mechanisms of action. Cultures were exposed for one week and two weeks (wk). Changes in pro collagen levels are reported relative to the values in MASH (red) and controls (blue). ** indicates *p *< 0.01 in heteroscedastic two‐tailed *t‐*test. Error bars represent SEM.

Fibrosis severity is the only histologic predictor of liver‐related morbidity and mortality in MASLD patients.^[^ ^46^ ^]^ To enable a patient‐centric drug discovery approach, we thus evaluated whether 3D MASH spheroids were susceptible to pharmacological modulation of fibrosis. Due to the extensive evidence of the role of TGFß signaling in liver fibrosis,^[^ ^47^ ^]^ we first evaluated the effects of the TGFß receptor inhibitor A8301 (**Figure** **3**a). A8301 treatment attenuated the fibrotic phenotype, as evidenced by a significant reduction of the activated stellate cell marker αSMA while maintaining stellate cell numbers (vimentin staining). Next, we tested the anti‐fibrotic effects of resmetirom and obeticholic acid (OCA), the only compounds that resulted in significant reductions of fibrosis in phase 3 randomized controlled trials (RCT). Our results indicate that pro‐collagen levels were reduced after five and seven days, but not after two days of treatment (Figure 3b). These effects are statistically significant and suggest that pro‐collagen secretion is reduced by 6–11% after one week of in vitro exposure (Figure 3c).

We then expanded the assessment to 14 candidates for MASH treatment currently under clinical development (Figure 3d). Among these, the metabolic modulators firsocostat, metformin, and aramchol, as well as the JNK1 inhibitor CC‐90001 showed significant dose‐dependent anti‐fibrotic responses. In contrast, other candidates that act primarily on extrahepatic tissues (FGF21, lanifibranor) or did not reduce fibrosis in the clinics (cenicriviroc, elafibranor, selonsertib) were negative. Using the dual PPAR‐α/δ agonist elafibranor as an example, we evaluated whether spheroid results could provide mechanistic insights at the systems level. Indeed, we found that elafibranor reduced steatosis without exhibiting positive effects on fibrosis (Figure S2a,b, Supporting Information). Transcriptomic analyses indicate that elafibranor resulted in distinct gene expression changes and gene set enrichment analyses showed significant enrichment in lipid catabolism and anti‐inflammatory processes without altering pathways related to collagen deposition or ECM remodeling (Figure S2c–e, Supporting Information). These results closely mirror the results of the respective RCT (NCT02704403) that showed anti‐steatotic and anti‐inflammatory effects without improvement of fibrosis. Combined, these results show that the MASH platform is accessible to phenotypic drug testing and the pharmacological benchmarking provides evidence for its translational value in accelerating patient‐centric drug discovery.

### 2.4 Drug Discovery Campaign Covering Chemical Modulators of DNA Damage Repair and Cellular Bioenergetics Identifies OGG1 as an Anti‐Fibrotic Target

**Figure 4:**
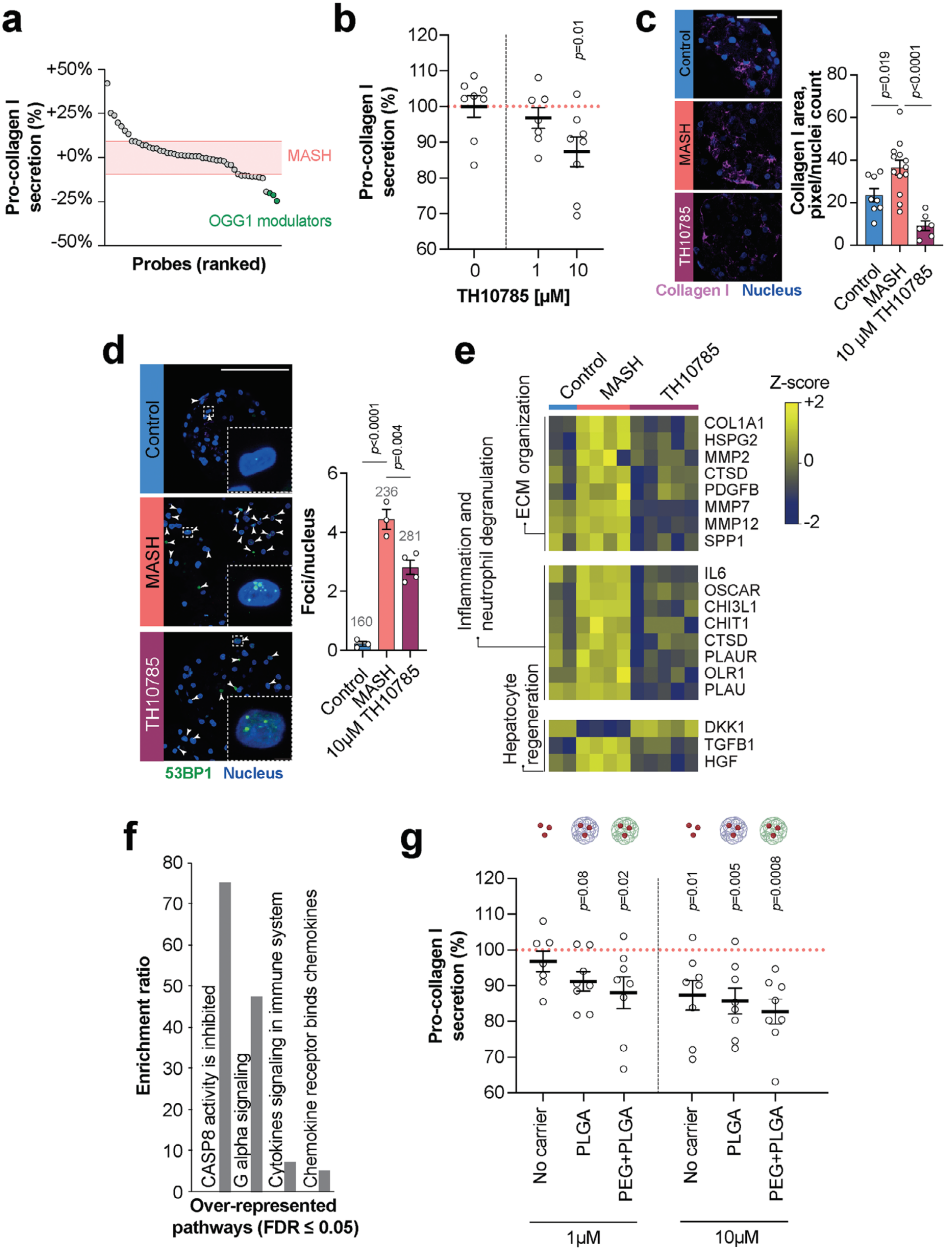
Drug discovery campaign for the prioritization of anti‐fibrotic small molecules targeting DNA damage repair. a) Relative pro‐collagen I secretion of MASH spheroids treated with a compound library targeting DNA repair, including mitochondrial and nuclear OGG1, and cellular bioenergetics. The top three hits are nuclear OGG1 modulators, including TH10785. The red shaded box indicates pro‐collagen secretion in the untreated MASH group ± SD (*n* = 10). b) Confirmation of the dose‐dependent anti‐fibrotic effects of TH10785 in independent replicate experiments. c) TH10785 results in a significant reduction of collagen I levels in MASH spheroids after 7 days of treatment. *n* = 6–13 sections. The total number of analyzed cells is shown in grey. Scale bar = 50 µm. d) TH10785 treatment for 7 days showed significantly reduced recruitment of 53BP1, a double‐strand DNA damage marker. Insets show a representative nucleus at higher magnification. The total number of nuclei analyzed for 53BP1 foci is shown in grey. Scale bar = 100 µm. e) Olink analysis of the secretome of 3D MASH cultures. TH10785 treatment resulted in significant downregulation of proteins involved in extracellular matrix (ECM) organization and neutrophil degranulation. f) Over‐representation analysis (ORA) of differentially abundant proteins between MASH and MASH + TH10785 groups. g) Encapsulation in polymeric nanocarriers improves TH10785 efficacy. Note that TH10785 in PEG‐PLGA carriers exert significant pro‐collagen reduction already at the lowest concentration (1 µm) tested. *p*‐values refer to heteroscedastic two‐tailed *t*‐tests for each group against MASH. All column plots are shown as mean ± SEM.

Oxidative stress due to reactive oxygen species plays a major role in MASH progression and has been linked to DNA damage and pathological polyploidization.^[^ ^48^ ^]^ To evaluate whether modulation of DNA damage repair and cellular bioenergetics would improve hepatic fibrosis, we used our model to evaluate a chemical series comprising 50 small molecule modulators of OGG1, SIRT1, and NAMPT. Three small molecules exhibited significant anti‐fibrotic effects, which all targeted OGG1 (**Figure** **4**a). These compounds included TH10785, a cellularly active organocatalytic switch that increases the enzymatic activity of OGG1 on apurinic sites by 20‐fold.^[^ ^49^ ^]^ The anti‐fibrotic effects of TH10785 were confirmed using secreted markers of hepatic fibrosis, as well as by IHC which showed a significant reduction of collagen I deposition (Figure 4b,c). Furthermore, 53BP1 staining indicates that TH10785 exposure significantly improved double‐strand DNA damage repair (Figure 4d).

To further probe the effect of TH10785, we performed targeted Olink proteomics of 142 secreted proteins. TH10785 treatment decreased levels of TGFB1 and COL1A1, as well as other factors involved in inflammation and neutrophil degranulation, including IL6, CHIT1, CHI3L1, and OSCAR compared to untreated MASH controls (Figure 4e). Overrepresentation analysis showed that CASP8 inhibition, Gα signaling as well as cytokine and chemokine signaling were the most significantly enriched pathways (Figure 4f). To prime the translational impact of the compound, we tested the efficacy of TH10785 with two well‐established nanocarrier formulations based on poly(lactic*‐co*‐glycolic acid) (PLGA) and polyethylene glycol‐functionalized PLGA (PEG‐PLGA). The drug was efficiently incorporated in the nanocarriers (encapsulation efficiency: 98.4 ± 1.1% for PLGA carriers, 89.4 ± 2.2% for PEG‐PLGA carriers). Negatively charged nanoformulations with hydrodynamic diameters <200 nm increased the anti‐fibrotic effect of TH10785, particularly at lower concentrations of the applied drug (Figure 4g). Together, these data show the utility of the MASH model as an integrative tool for compound prioritization, demonstrate its compatibility with nanoparticle‐based drug delivery, and highlight OGG1 as a promising anti‐fibrotic target.

### 2.5 Chemogenomic Screening in Patient‐Derived MASH Cultures Reveals Novel Pharmacologically Accessible Targets

**Figure 5:**
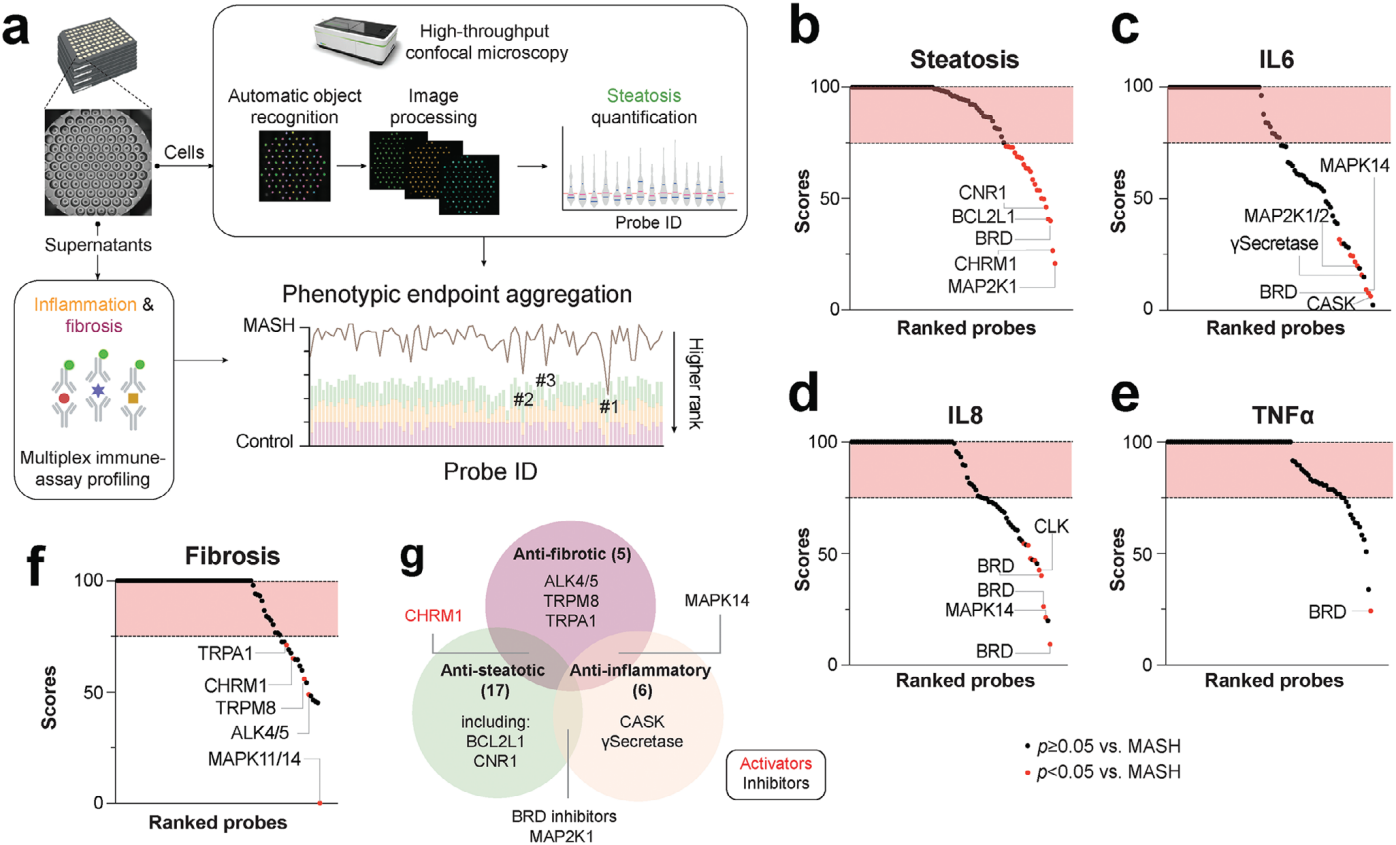
Chemogenomic screening based on multiplexed phenotypic readouts identifies novel druggable targets. a) Schematic of the primary screening setup for the evaluation of 92 chemical probes. The screening was conducted in 96‐well ultralow attachment microwell arrays. Compound effects on steatosis were evaluated using automated high‐throughput confocal microscopy, whereas effects on inflammation and fibrosis were evaluated using multiplexed immune assays for IL6, IL8, TNFα and pro‐collagen I. b–f) Waterfall plots showing reduction of steatosis (b), IL6 (c), IL8 (d), TNFα (e), and pro‐collagen I (f) secretion. Only compounds that reduced the respective endpoint by >25% (outside the red‐shaded interval) and where the reduction was statistically significant (*p *< 0.05; red dots) were considered as hits. g) Venn diagram summarizing the identified target proteins. Activators are shown in red; inhibitors are shown in black.

To identify novel MASH targets, we leveraged a diverse library of 92 chemical tool compounds provided by the Structural Genomics Consortium (SGC; https://www.thesgc.org/). The probes exhibit potency on the intended target <100 nm, have cellular on‐target activities <1 µm, are at least >30‐fold selective within the target family and have been extensively profiled for off‐target effects outside the target family.^[^ ^50^ ^]^ The set is enriched for functional modulators of kinases, enzymes, G‐protein coupled receptors (GPCRs), and ion channels and has been comprehensively evaluated in human tissue models for multiple endpoints, including hepatotoxicity.^[^ ^51^ ^]^ Here, we performed a high‐content screen in which responses at the level of lipid accumulation, pro‐inflammatory cytokine, and pro‐collagen Ia1 secretion were quantified in a parallelized multiplexed assay (**Figure** **5**a).

Most hits were identified for steatosis where a total of 23 compounds significantly (*p *< 0.05) reduced intracellular triglyceride levels by >25% (Figure 5b). These included hits for six kinases, three ion channels, and four GPCRs. For cytokines, we identified 10, 9, and 1 hits for IL6, IL8, and TNFα, respectively (Figure 5c–e). The hit compounds showed substantial overlap between the cytokines with various BRD (GSK‐973, GSK‐620, GSK‐046) and MAPK14 (FS‐694) inhibitors significantly reducing multiple tested cytokines. For fibrosis, a total of 5 hits were detected, which included an inhibitor of ALK4/5 (TP‐008), a well‐known anti‐fibrotic target (Figure 5f). Further hits targeted, MAPK11/14 the ion channels TRPA1 and TRPM8, as well as the GPCR CHRM1. Hits did not overtly impact cell viability (Table S7, Supporting Information) and were replicated in independent concentration‐response experiments using a >100‐fold less potent control compound with the same chemical scaffold as a comparator, where available (Figure S3, Supporting Information). The results confirmed that PQCA, a positive allosteric modulator of the muscarinic M_1_ receptor (CHRM1), significantly reduced steatosis and fibrosis, whereas no significant effects were observed with the inactive paired control. Similar results were observed for the ALK4/5 inhibitor TP‐008. Furthermore, concentration‐dependent inhibition of steatosis and fibrosis was replicated for selumetinib (MAP2K1 inhibitor), MK‐2206 (AKT inhibitor), and PF‐05105679 (TRPM8 inhibitor). Considering target redundancy, we identified a total of 24 protein hits of which most (*n* = 20; 83%) were positive for a single endpoint, whereas CHRM1, MAPK14, MAP2K1, and BRDs exhibited combinatorial effects (Figure 5g). Overall, these results showed that chemogenomic screening in patient‐derived 3D MASH cultures can indicate novel druggable targets involved in disease‐relevant endpoints.

### 2.6 The CHRM1 – TRPM8 Signaling Axis Contributes a Novel Disease Module in Hepatic Fibrosis

**Figure 6:**
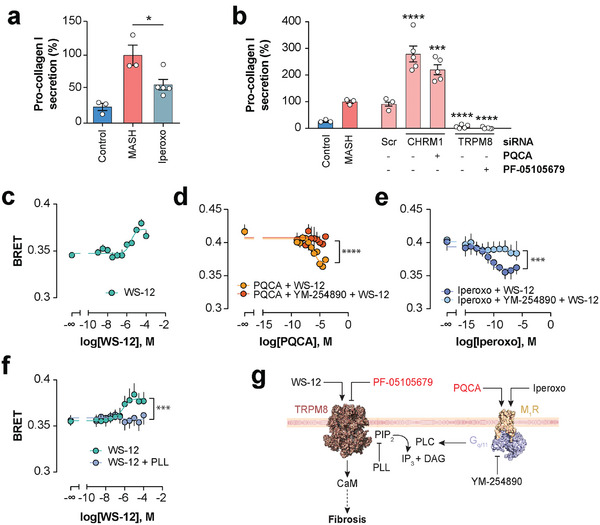
CHRM1 and TRPM8 functionally interact to regulate hepatic fibrosis. a) Anti‐fibrotic effects of PQCA are confirmed using the muscarinic receptor superagonist iperoxo. *n* = 3–5 replicates per group. b) Effects of knock‐down of CHRM1 and TRPM8 on pro‐collagen I secretion in MASH cultures. Note that PQCA, the anti‐fibrotic hit targeting CHRM1, is a positive allosteric modulator and the knock‐down thus results in an opposite pro‐fibrotic effect. *n* = 3–5 replicates per group. c–f) Bioluminescence resonance energy transfer (BRET) assays monitoring the ligand‐dependent recruitment of mNeonG‐CaM to TRPM8‐Nluc. c) Activation of TRPM8 using the specific TRPM8 agonist WS‐12. *n* = 4. d) Concentration‐response curve of PQCA in the presence of WS‐12 (orange) or WS‐12 and the G_q/11_ antagonist YM‐254890 (red). *N* = 3. e) Concentration‐response curve of iperoxo in the presence of WS‐12 (dark blue) or WS‐12 and the G_q/11_ antagonist YM‐254890 (cyan). *n* = 4. f) Concentration‐response curve of WS‐12 with (blue) or without (teal) the PIP_2_ scavenger PLL. *n* = 5. g) Molecular model depicting the interactions between the muscarinic receptor CHRM1 (M_1_R) and the calcium channel TRPM8 in human MASH. Compounds in red denote hit probes from the chemogenomic screen. CaM = calmodulin; DAG = diacylglycerol; IP_3_ = inositol‐1,4,5‐trisphosphate; PIP_2_ = phosphatidylinositol 4,5‐bisphosphate; PLC = phospholipase C. *, **, ***, **** indicate *p *< 0.05, *p *< 0.01, *p *< 0.001, and *p *< 0.0001, respectively, in heteroscedastic two‐tailed *t*‐tests compared to MASH (panels a and b) or *F*‐tests comparing the fits of the respective curves (panels c–f). Error bars indicate SEM.

Among the identified hits, the TRPM8 ion channel had previously been shown to be regulated by heterotrimeric G proteins.^[^ ^52^ ^]^ To evaluate whether GPCR‐based regulation of channel function could constitute a novel molecular mechanism in hepatic fibrosis, we first aimed to further corroborate the pharmacological results of the PQCA tool compound. Treatment with the established pan muscarinic receptor superagonist iperoxo significantly alleviated fibrosis (*p *= 0.01; **Figure** **6**a). Next, we employed an orthogonal genetic approach. Consistent with the anti‐fibrotic effect of pharmacological CHRM1 activation, we found that siRNA‐based knock‐down of CHRM1 resulted in an amplification of fibrosis (*p *< 0.001), irrespective of the co‐exposure with PQCA (Figure 6b). In contrast, silencing of TRPM8 led to a significant decrease of pro‐collagen levels in MASH patient‐derived spheroids, again confirming the results from small molecule‐based inhibition. The results of the knock‐down experiments thus confirm the anti‐fibrotic effects of M_1_R activation and TRPM8 inhibition.

To probe whether CHRM1 and TRPM8 functionally interact, we adopted a molecular biosensor strategy based on BRET that monitors TRPM8 activation via calmodulin (CaM) coupling during gating.^[^ ^53^ ^]^ First, we demonstrate that activation of the TRPM8 channel by WS‐12 resulted in a concentration‐dependent increase in BRET, as expected (Figure 6c). Strikingly, activation of CHRM1 via either iperoxo or PQCA significantly inhibited WS‐12‐mediated TRPM8 activation, demonstrating a clear functional interaction between CHRM1 and TRPM8 (Figure 6d,e). Co‐exposure with the G_q/11_ inhibitor YM‐254890 abrogated the inhibitory effect of CHRM1 activation, demonstrating that G_q/11_ is the principal G protein transducer along the CHRM1‐TRPM8 axis. We hypothesized that G_q/11_ might impact TRMP8 activity via controlling phospholipase C (PLC) activity.^[^ ^54^ ^]^ We thus used PLL to sequester phosphatidylinositol 4,5‐bisphosphate (PIP_2_), the substrate of PLC and found that co‐treatment abolished WS‐12 mediated TRPM8 activation (Figure 6f). Combined, these results lend strong support for a model in which CHRM1 activation results in G_q/11_ and subsequent activation of PLC (Figure 6g). The resulting depletion of PIP_2_ inactivates the TRPM8 channel, in agreement with structural data demonstrating that channel opening requires allosteric PIP_2_ binding.^[^ ^55^ ^]^ A combination of chemogenomic screening in patient‐derived 3D tissue models with mechanistic molecular follow‐ups thus identified a new MASH disease module and demonstrated the potential of scalable organotypic tissue models for systematic drug discovery.

## 3 Discussion

The development of drugs against MASH has been a substantial challenge with more than two decades of clinical failures despite promising results in preclinical animal models. The recent approval of resmetirom constitutes an important breakthrough for MASH therapy; however, less than 30% of patients benefitted from this treatment,^[^ ^56^ ^]^ which means that a considerable need for new therapeutic agents remains. The main problems pertaining to MASH drug discovery and development are arguably the complexity and inter‐individual variability of the disease, which jointly contribute to a still limited understanding of its molecular mechanisms. Previous MASH studies were mostly done in rodents; however, this approach is significantly hindered by species differences.^[^ ^11^ ^]^ The need to faithfully emulate a chronic disease that typically takes decades to manifest in humans, within a few months due to the limited rodent lifespan constitutes a main obstacle. Furthermore, metabolic comorbidities, such as obesity and type 2 diabetes, as well as contributing factors, such as a sedentary lifestyle, are difficult to mimic in experimental animal models. The development of pathophysiologically relevant patient‐derived 3D models promises to close an important gap in the methodological tool kit for studying MASH.

Previous in vitro models mostly employed hepatic cell lines or used passaged primary cells from donors without an established MASH diagnosis, which mandated the addition of cytokines or lipopolysaccharides to illicit hepatocyte stress and fibrosis.^[^ ^57^, ^58^, ^59^, ^60^ ^]^ While these models can be high‐throughput compatible, they lack the accurate emulation of relevant disease phenotypes. Organ‐on‐a‐chip systems in which hepatocytes and NPCs are cultured in 2D on opposing sides of a permeable membrane have shown promise in recapitulating MASLD phenotypes and providing new insights into the therapeutic effect of candidate drugs.^[^ ^61^ ^]^ However, they are generally low‐throughput and, at present, can only be used to study acute phenomena for <3 days.^[^ ^62^ ^]^ Alternatively, models based on induced pluripotent, embryonic, or adult hepatic stem cells can be used to generate hepatic organoids at scale. These systems emulate fatty acid‐induced steatosis and inflammation; moreover, evaluation of organoid stiffness can be used as a proxy for fibrosis.^[^ ^63^, ^64^, ^65^ ^]^ Hepatic organoids can mimic the effect of genetic factors and CRISPR profiling of 35 genes has recently been used to identify FADS2 as an important determinant of hepatic steatosis.^[^ ^66^, ^67^ ^]^ However, the use of organoids for MASH drug development is hampered by multiple factors. First, organoid cultures are heterogeneous, resulting in large intra‐experimental variability, which hinders large‐scale screening. Second, they require predefined matrix compositions, which alter the endogenous regulation of hepatic scarring. Third, as organoids develop from few cells using protocols that foster proliferation and controlled differentiation, they cannot reflect the exposure history of mature hepatocytes, which can have lifespans of up to 5 years.^[^ ^68^ ^]^

The MASH model presented here is the first to be established from fully mature patient‐derived cells and thus captures the environmental exposure history of the diseased cells. Consequently, MASH spheroids recapitulate steatosis, inflammation, and fibrosis, as well as the associated molecular signatures at the transcriptomic, proteomic, and lipidomic levels. Specifically, we identify upregulation of cholesterol biosynthesis, which occurs concomitantly with the induction of MLX activity that is known to increase glycolysis and lipogenesis. Additionally, the increase in perilipin (PLIN2) at the proteomic level as well as DAGs and TAGs underscores the increase in de novo lipogenesis and aligns with hepatic steatosis in MASH. The increase in cholesterol biosynthesis can cause alterations in the dynamics of the membranes resulting in increased oxidative injury and an aggravation of hepatic inflammation.^[^ ^69^ ^]^ This, alongside with elevations of acetylcarnitine lipids reflects mitochondrial dysfunction in lipid metabolism, impairing beta‐oxidation. Moreover, the increase in activity of the transcription factors RELA and IRF1 points to a pro‐inflammatory environment. Increased levels of the SNAT1 transporter suggest altered nutrient sensing, potentially further promoting the accumulation of toxic lipids and exacerbating liver stress. Furthermore, the upregulation of ECM organization and collagen deposition pathways denotes active fibrogenesis and pathological remodeling of the microphysiological hepatic niche.

As mature liver cells in the spheroid model are long‐lived and, as in vivo, hardly proliferate,^[^ ^70^ ^]^ the different 3D microtissues are highly homogeneous, rendering the system compatible with highly parallelized multiplex evaluations with mid‐to‐high throughput. Specifically, our liver 3D spheroids cultures are cultured in 96‐ or 384‐well plate format and up to 60 plates can be run in parallel by a single experimentalist. This enables the testing of >20 000 spheroids or, considering 8–16 replicates per condition, ≈2000 compounds in a single experiment.

For experimental planning, it is important to consider that, as any in vitro model, the 3D MASH model presented here is reductionistic. This entails that the anti‐MASH effect of compounds that act primarily on extra‐hepatic cells and tissues, such as SGLT2 inhibitors or GLP1R agonists, cannot be meaningfully studied. Integration of patient‐derived cholangiocytes^[^ ^71^ ^]^ or liver sinusoidal endothelial cells,^[^ ^72^ ^]^ which are thought to play important roles in inflammatory signaling, inflammation, and hepatic ECM remodeling, constitutes an area of further development. However, the reductionistic approach outlined here also offers opportunities, for instance, by uncoupling direct hepatic effects from contributions of dysfunctional adipose tissue, pancreas, or skeletal muscle, which are commonly observed in metabolic syndrome. Furthermore, we note that more work is required to characterize the inter‐individual variability of spheroid cultures. This includes the analysis of non‐parenchymal cell composition, functionality, as well as their stability in culture over time. We previously showed that subsets of liver macrophages can have significant effects on cellular stress in steatotic spheroids,^[^ ^73^ ^]^ emphasizing that these cells retain their functional heterogeneity. However, sufficiently powered profiling of different subpopulations across MASLD and MASH donors as well as investigations into their functional contributions are needed. In addition, it will also be important to evaluate differences in drug response by comparing spheroids generated from donors of different ages, sexes, metabolic characteristics, and MASH disease stages.

The chemogenomic pilot screen presented here resulted in the identification of multiple anti‐steatotic, anti‐inflammatory, and anti‐fibrotic hits. Of these, five were already validated in independent replication experiments and via the use of inactive chemical analogs, serving as potential starting points for future drug development projects. Among the top hits was CHRM1. While the receptor had not been previously implicated as a potential target in MASH, multiple lines of evidence support its role in steatosis and fibrosis. First, dietary deficiency in choline, the precursor of the CHRM1 ligand acetylcholine, is linked to the development and progression of MASH in both experimental animals and human association studies.^[^ ^74^ ^]^ Furthermore, knock‐out mice in which *Pemt* or *Bhmt2*, the key genes of choline biosynthesis, were ablated, developed MASLD and MASH.^[^ ^75^, ^76^ ^]^ These findings aligned with results from our MASH model where PEMT and BHMT2 were significantly downregulated in MASH compared to Control (PEMT; *p *= 0.04; BHMT2; *p *= 0.02). Additionally, the low levels of phosphatidylcholines in MASH patient livers^[^ ^77^ ^]^ are also identified as significant alterations in MASH spheroids, corroborating that disease‐relevant lipidomic perturbations are also carried over into the in vitro setting. Seminal studies from 1949 furthermore showed that choline supplementation could prevent fatty liver and fibrosis in rat models of ethanol‐ or sucrose‐induced liver injury;^[^ ^78^ ^]^ the underlying mechanism however has remained elusive. Our results suggest that choline might exert its protective effect via inhibition of excessive TRPM8‐mediated calcium influx, which is of central importance for the orchestration of metabolic control in the liver. Elevated cytosolic calcium levels activate the Calcium/Calmodulin‐dependent protein kinase CaMKK2,^[^ ^79^ ^]^ which phosphorylates and thereby stimulates AMPK,^[^ ^80^ ^]^ a central regulator of metabolic liver homeostasis. Furthermore, chronically elevated cytosolic calcium levels can spill over into the mitochondria, resulting in the excessive activation of pyruvate dehydrogenase, as well as the increased production of reactive oxygen species and mitochondrial stress.^[^ ^81^ ^]^

## 4 Conclusion

The FDA Modernization Act 2.0 has formalized the realization that emerging organotypic human model systems can facilitate the development of more translatable findings compared to animal testing.^[^ ^17^ ^]^ Particularly, in light of expanding chemical probe programs, such as Target2035,^[^ ^82^ ^]^ which aspire to develop specific pharmacological modulators for every protein in the human proteome, we believe that the presented results can provide an important proof‐of‐concept that phenotypic screening of disease‐relevant endpoints in patient‐derived cultures can identify novel human‐specific disease modules. This information might open up new avenues for drug development and can act as a catalyst to develop similar patient‐centric approaches for other pathologies, particularly those with complex and incompletely understood molecular underpinnings.

## 5 Experimental Section

### Cell Culture

Spheroid culture of cryopreserved primary human liver cells was conducted as previously described^[^ ^23^ ^]^ with relevant modifications detailed below. The demographics and available medical history of the donors are shown in Table S1 (Supporting Information). Cells were acquired from donor liver tissue at Karolinska University Hospital according to ethical protocol (2014/1561‐32) or were commercially acquired from BioIVT (USA) and LifeNet Health (USA). All samples were obtained upon informed consent from each donor or the subject's legally authorized guardian, which was reviewed and approved by the appropriate regulatory authorities in accordance with the HHS regulations for the protection of human subjects (45 CFR §46.116 and §46.117) and Good Clinical Practice (ICH E6). Liver cells were seeded at a PHH: NPC ratio of 6:1 into multi‐well 96‐well plates (Elplasia, Corning) with a total of 40 000 cells well^−1^ in culture medium (William's E medium containing 11mm glucose, 100 nm dexamethasone, 10 ng mL^−1^ (control) or 10 µg mL^−1^ insulin (MASH), 5.5 mg L^−1^ transferrin, 6.7 µg L^−1^ selenite, 2 mm *L‐*glutamine, 100 U mL^−1^ penicillin, 0.1 mg mL^−1^ streptomycin) supplemented with 10% fetal bovine serum (FBS). After spheroid formation, FBS was phased out and cells were maintained in a control medium or medium supplemented with albumin‐conjugated free‐fatty acids (FFA), comprised of 240 µm palmitic acid and 240 µm oleic acid. Culture media were changed every 2–3 days.

### Flow Cytometry

Flow cytometry staining was performed as previously described.^[^ ^83^ ^]^ Cells were stained with antibodies targeting CD3 (BUV661, clone UCHT1, BD Biosciences, 1/50), CD14 (BUV805, clone M5E2, BD Biosciences, 1/25), HLADR (BV785, clone L243, Biolegend, 1/25), CD56 (BUV563, clone NCAM16.2, BD Biosciences, 1/200), CD16 (BUV496, clone 3G8, BD Biosciences, 1/25), CD161 (biotin, clone 191B8, Miltenyi, 1/50), streptavidin BV650, BD Biosciences, 1/200), TCRva7.2 (PE, clone 3C10, Biolegend, 1/50), CD19 (BV510, clone SJ25C1, BD Biosciences, 1/100), CD163 (AF647, clone GHI/61, BD Biosciences, 1/100), CD206 (BB515, clone 19.2, BD Biosciences, 1/25), CD4 (BV750, clone SK3, BD Biosciences, 1/50), CD8 (BV605, clone RPA‐T8, Biolegend, 1/200), KIRs (PECy5.5, combination of clones GL183 in 1/50 and EB6B in 1/20 from Beckman Coulter), NKG2A (PECy7, clone Z199, Beckman Coulter, 1/100), CD45 (AF700, clone HI30, Biolegend, 1/200) and FcR block (Miltenyi, 1/25). For dead cell exclusion, a fixable LIVE/DEAD Yellow dead cell stain kit (Life Technologies) was used. Data were acquired on a BD LSR Symphony and analyzed in FlowJo V10.

### Viability Assay

Intracellular ATP was used as a proxy for cellular viability and was measured by luminescence‐based ATP assay (CellTiter Glo; Promega) according to the manufacturer's instructions with 0.1% SDS added to facilitate lysis.

### Steatosis Assessment

For screening applications, spheroids were fixed with 0.4% (v/v) methanol‐free formaldehyde, washed with PBS, and stained overnight with Hoechst33342 and NileRed. Imaging was performed on a high‐throughput confocal microscope (Opera Phenix, Revvity) with a 10× objective. Twenty‐five image fields across 10 z‐stacks were obtained for each well. Spheroid roundness, area, and location were extracted using the PhenoLOGIC algorithm in Harmony and steatosis was automatically quantified for each spheroid as the ratio of triglyceride to nuclear signal. For validation experiments, lipid accumulation was measured using the AdipoRed Assay (Lonza) following the manufacturer's instructions.

### Immunoassays

Pro‐collagen and various of its neoepitopes were promising non‐invasive biomarkers for liver fibrosis.^[^ ^84^ ^]^ Here, pro‐collagen Ia1 levels were measured in culture supernatants using the Human Pro‐collagen I alpha 1 DuoSet ELISA (R&D Systems; DY6220‐05) following the manufacturer's protocol. For cytokine quantifications, multiplex bead‐array immunoassays were performed as previously described.^[^ ^85^ ^]^ In brief, the Human High Sensitivity Cytokine Magnetic Panel A (customized selection; IL6, IL8, and TNFα) was purchased from Bio‐Techne. Standards and diluted cell culture supernatants were mixed with the beads and incubated for 3 h on an orbital shaker at 800 rpm in the dark. The plates were washed with a magnetic plate washer and then incubated for 1 h with biotinylated detection antibodies followed by 30 min incubation with PE‐conjugated streptavidin solution. Fluorescence intensity was measured using a Bio‐Plex 200 system (Bio‐Rad) and each analyte was analyzed with the Bio‐Plex Manager software (v6.2) using a 5‐parameter regression algorithm.

### Immunofluorescence

Liver spheroids were fixed in 4% paraformaldehyde overnight, followed by incubation in 30% sucrose‐PBS at 4 °C until the microtissues sank. For cryo‐mount embedding, spheroids were transferred into OCT molds, embedded in Tissue‐Tek OCT compound (Sakura, The Netherlands), and frozen in an isopropanol dry ice bath. Subsequently, microtissues were sectioned at a thickness of 10µm using a CryoStar NX70 cryostat (Epredia). Sections were washed twice with PBS and then blocked with PBTA buffer (5% BSA, 0.25% Triton X‐100, 0.01% NaN_3_ in PBS) for 2 h at room temperature. Subsequently, sections were incubated overnight at 4 °C with monoclonal primary antibodies against αSMA (1:200 ab7817, Abcam) or vimentin (1:200 ab92547, Abcam). For detection, sections were incubated with Alexa Fluor‐conjugated secondary for 2 h at room temperature and, after a final wash, the slides were mounted in Prolong Gold Antifade mounting reagent with DAPI (Thermo Fisher Scientific). Samples were imaged on an LSM880 confocal microscope (Zeiss).

### Immunohistochemistry

Liver spheroids were embedded in paraffin and sectioned (3 µm) using an RM2255 microtome (Leica). Sections were stained with anti‐COL1A1 (ab34710, Abcam) and anti‐CD68 (sc‐70761, Santa Cruz). For detection, DAKO Real EnVision Detection System (Agilent) with peroxidase/DAB+ colorimetric was used and as counterstain hematoxylin was used. The stained slides were imaged with an Olympus VS120 slide scanner with OlyVIA software v3.3 (Olympus).

### DNA Damage Assay

Spheroids were fixed and embedded as described above. Spheroid cryosections (8 µm) were stained for 53BP1 (1:1000 ab36823, Abcam) and then counter‐stained with DAPI in mounting media (Invitrogen, P36935). Images were obtained with a 63× oil immersion objective with Leica Stellaris 5 LIA. 53BP1 foci and nuclei were identified and counted using an in‐house pipeline, which consisted of automatic contrast enhancement, denoising by intensity filtering, segmentation by watershed, and particle analysis in Fiji (ImageJ).

### siRNA Knock‐Down Experiments

Primary human hepatocytes were transfected with siRNA against CHRM1 (M‐005462‐01‐0005; Dharmacon) and TRPM8 (M‐006517‐01‐0005; Dharmacon) at final concentrations of 250 nm using RNAiMAX (Invitrogen) according to the manufacturer's instructions. The respective siRNA was mixed with serum‐free Opti‐MEM medium, and the mixture was incubated at room temperature for 20 min. Subsequently, the siRNA was added to the cell suspension before distribution into ULA plates for further culture.

### RNA‐Sequencing and Transcriptomic Data Analysis

Total RNA was isolated using a Qiazol lysis reagent (QIAGEN). Samples were processed using the NEBNext Ultra II Directional RNA Library Prep Kit (NEB) and sequenced on a NovaSeq6000 instrument (Illumina) at GenomeScan BV (Leiden, Netherlands). Raw data were processed using the RTA3.4.4 pipeline and Bcl2fastq (v2.20) conversion software (Illumina). Expression of genes that were detected in at least 25% of samples was analyzed using Qlucore Omics Explorer v3.7 (Lund, Sweden). Reactome pathway analyses were carried out using WebGestalt.^[^ ^86^ ^]^ Gene set enrichment analysis (GSEA) was performed using GSEA software v4.3.2 against MSigDB. Transcription factor activity profiles were calculated using ISMARA.^[^ ^87^ ^]^ This established tool inferred transcription factor activities based on a linear model with a Bayesian procedure where a Gaussian prior on the respective motif activities was used to avoid overfitting. To compare expression patterns between spheroids and MASH biopsies, RNA‐Seq data was compared from MASH spheroids with transcriptomic datasets from six MASH case‐control cohort studies (GSE173735, GSE105127, GSE147304, GSE126848, GSE135251 and GSE162694). Transcriptomic data from control spheroid cultures were used as a reference.^[^ ^88^ ^]^ All fastq files were processed identically by quantification in Salmon using GRChg38.14 as the reference transcriptome, followed by batch effect elimination using the ComBat^[^ ^89^ ^]^ function of the sva package in R (v4.3.3).

### Proteomics and Phosphoproteomics

Cell pellets were lysed in 5% SDS, 5 mm tris(2‐carboxyethyl)phosphine, 10 mm chloroacetamide, 100 mm Tris pH 8.5 heated at 95 °C and mixed in a thermo‐shaker at 1000 rpm and 95 °C for 10 min. Lysates were sonicated using a probe sonicator, the protein amounts were measured using a bicinchoninic acid assay, and an equal amount of protein in each sample was used for sample preparation. Proteins were digested overnight using Protein Aggregation Capture^[^ ^90^ ^]^ in a KingFisher Flex robot (Thermo Fisher Scientific) with 2:1 MagReSyn Hydroxyl beads to protein ratio (w/w). Acetonitrile (ACN) was added to a concentration of 20% and peptides were labeled with TMTpro 16‐plex Label Reagent (Thermo Fisher Scientific) at 1:4 protein to TMT label ratio. The reaction was quenched by 0.5% hydroxylamine (Sigma‐Aldrich) for 15 min at RT. Samples were pooled, acidified using TFA, desalted using SepPak, and dried using a speedvac. The resulting multiplexed TMT‐labeled peptide mixtures were resuspended in 50 mm ammonium bicarbonate, split in two for separate sample preparations for full proteome and phosphoproteomics analyses and each sample was fractionated by offline high pH reversed‐phase chromatography using Acquity CSH C18 1.7 µm × 1 mm × 150 mm column (Waters) connected to an UltiMate 3000 high‐performance liquid chromatography (HPLC) system (Thermo Fisher Scientific). The instrument was operating at 30 µl min^−1^ with mobile phases consisting of buffer A (5 mm ammonium bicarbonate) and buffer B (100% ACN). Phosphopeptide enrichment was carried out on a KingFisher Flex robot (Thermo Fisher Scientific) using TiIMAC‐HP beads (MagReSyn, Resyn Biosciences) as previously described.^[^ ^91^ ^]^ Fractions enriched for phosphopeptides were acidified using TFA and loaded onto Evotips (Evosep) according to the manufacturer's protocol.

All samples were analyzed by LC‐MS/MS using the Evosep One liquid chromatography system. The phosphopeptides fractions were analyzed with the 20SPD (WHISPER) gradient and an IonOpticks Aurora column (15 cm‐75 µm‐C18 1.6 µm) coupled to an Orbitrap Exploris 480 (Thermo Fisher Scientific). The full proteome fractions were analyzed using a 30SPD gradient with an Evosep Endurance column (15 cm × 150 µm, 1.9 µm) coupled to a Lumos Fusion Orbitrap (Thermo Fisher Scientific). On the Exploris 480, the spray voltage and RF lens were set to 1.8 kV and 30% while they were set at 2 kV and 40% on the Lumos, and the heated capillary temperature at 275 °C for both instruments.

Raw files from full proteome and phosphoproteomics analyses were searched separately with MaxQuant (v2.1.4.0) using the Andromeda search engine. The phosphopeptide quantification data were collapsed to site‐level information using the peptide collapse plugin from the Perseus platform.^[^ ^92^ ^]^ Raw intensities were log2 transformed and normalized across a TMT set using median‐based normalization. Kinase‐substrate enrichment analysis was performed using RoKAI.^[^ ^93^ ^]^

### Olink Targeted Proteomic Discovery Panel

Targeted proteomic profiling of culture supernatants was performed with proximity extension assay technology using the Target 96 Inflammation, Cardiovascular II, and Cardiovascular III panels (Olink). Analyses were performed according to the manufacturer's instructions. Data were presented as normalized log2‐scaled protein expression (NPX) and proteins with NPX below the lower limit of detection in more than half of the samples were excluded.

### Lipidomics

Lipids were extracted as previously described^[^ ^94^ ^]^ with modifications as follows: The MMC solvent was spiked with the SPLASH mix internal standard supplemented with d7‐sphinganine, d7‐sphingosine, dihydroceramide, ceramide, deoxydihydroceramide, deoxyceramide and glucosylceramides (Avanti Polar Lipids). An XSelect CSH C18 column (100 mm × 2.1 mm, 2.5 µm particle size, Waters Corp.) and an Exion UHPLC pump (AB Sciex) were used to separate the lipids. Mobile phase A was composed of acetonitrile/water (60:40, v/v) with 10 mm ammonium formate and 0.1% formic acid, while mobile phase B was composed of isopropanol: ACN (90:10, v/v) with 10 mm ammonium formate and 0.1% formic acid. Chromatography was performed at a flow rate of 400 µl min^−1^ and a constant column temperature of 50 °C. The column was initially equilibrated with 40% B, then increased to 43% B over 2 min, to 50% B at 2.1 min, to 54% B at 12 min, to 70% at 12.1 min, to 99% B at 18 min, and finally re‐equilibrated with 40% B for an additional 2 min. A QTRAP 6500+ mass spectrometer in MRM acquisition mode (AB Sciex) was used for mass spectrometry analysis. Data was analyzed using the Skyline software package and the MetaboAnalyst Suite.^[^ ^95^ ^]^

### Bioluminescence Resonance Energy Transfer

TRPM8‐Nluc and mNeonG‐CaM were kindly provided by Yann Percherancier (Université de Bordeaux, France). M_1_R was purchased from cDNA.org (Bloomsburg University, PA, USA). All plasmid constructs were verified by Sanger sequencing. HEK293 cells were transfected with TRPM8‐Nluc, mNeonG‐CaM, and M_1_R using linear polyethyleneimine and seeded in white 96‐well plates (3.5 × 10^4^ cells well^−1^). Prior to BRET measurements, cells were incubated with coelenterazine h (10 min). Plates were read on a Spark multimode microplate reader (Tecan) equipped with a double monochromator system to measure the Nluc/mNeonG donor–acceptor pair in the calmodulin recruitment assay [445–485 nm (donor) and 505–560 nm (acceptor)].

### OGG1 Modulator Screening Campaign

In these efforts to improve DNA repair and cellular bioenergetics in disease, a database of small molecules covering cellular targets, including OGG1, SIRT1, and NAMPT was built. A primary screening set of 50 compounds was selected, which were synthesized as reported^[^ ^96^, ^97^ ^]^ or obtained commercially. After confirmation of identity by LC/MS, compounds were stored as aliquots either as solid or as 10 mm solutions in DMSO.

### Nanocarrier Synthesis and Analysis

PLGA and PEG‐PLGA nanocarriers with TH10785 payload were prepared by double emulsion solvent evaporation according to previous reports.^[^ ^98^ ^]^ Briefly, 200 µl of tris(hydroxymethyl)aminomethane‐buffer was added to a solution of the respective polymers in chloroform. After the primary emulsion was produced via sonication (30%, 60 s; Vibra Cell, VCX750, Sonics & Materials, Inc.), the secondary emulsion was prepared by dropwise addition of the primary emulsion to 6 ml of Tween‐80 in ultrapure water (1.67%, v/v) under sonication (40%, 120 s). Subsequently, the chloroform was evaporated under reduced pressure to yield solid nanocarriers. Formulations containing the active pharmaceutical ingredient were produced accordingly, with the drug dissolved in the chloroform phase. To separate freely dissolved drug molecules from the formulation, the nanocarrier dispersion was washed three times via centrifugation (21 500 g, 20 min, 4 °C), followed by re‐dispersion in ultrapure water. The resulting nanocarrier dispersions were stored under the exclusion of light at 8 °C until further use. Hydrodynamic diameters and ζpotentials were determined via dynamic light scattering (Zetasizer NanoZS, Malvern Panalytical). Drugloading was determined by analysis of the concentration of freely dissolved drugs in the supernatant collected during the first washing step. Analyses were performed via HPLC (Dionex UltiMate 3000, Thermo Fisher Scientific) on a 100 mm RP‐18 column (BDS Hypersil C18, 100 × 4.6 mm, 3 µm particle size, Thermo Fisher Scientific) using a mobile phase of ACN: water (0.1% TFA, v/v) 10–95% in 3min.

### Chemogenomic Screening

The effect of 92 chemical probes on steatosis, inflammation, and fibrosis was evaluated using the assays described above. The screening library consisted of the donated chemical probe (DCP) set, which constituted a panel of high‐quality chemical tools with high on‐target potency (<100 nm), high selectivity (>30‐fold within the target family), and extensive off‐target profiling.^[^ ^50^ ^]^ Ten thousand times concentrated stock solutions were prepared in DMSO and diluted to 1× in FFA‐supplemented culture medium prior to dosing. The used probes, their targets, exposure concentrations, and mechanisms of action are provided in Table S2 (Supporting Information).

### Statistical Analyses

Quantitative data were presented in violin plots spanning the interquartile range with the mean in the center and box plots or bar plots showing the mean ± standard error of the mean (SEM). Statistical comparisons between the two groups were conducted using unpaired two‐tailed heteroscedastic *t‐*tests. Multiple groups were compared using one‐way ANOVA tests with Dunnet's post‐hoc correction for multiple comparisons. Statistical analyses were done using Prism (GraphPad). All data are shown, and no outlier removal was performed. Results were considered significant if *P* ≤ 0.05.

## Conflict of Interest

VML is the co‐founder, CEO, and shareholder of HepaPredict AB, as well as the co‐founder and shareholder of Shanghai Hepo Biotechnology Ltd. The other authors declare no conflict of interest.

## Author Contributions

S.Y. and A.M.K. contributed equally to this work and share first authorship.

## Supporting information



Supporting Information

Supporting Figure and Table

## Data Availability

The data that support the findings of this study are available from the corresponding author upon reasonable request.
