# Supplementary material for: Chemogenomic Screening in a Patient‐Derived 3D Fatty Liver Disease Model Reveals the CHRM1‐TRPM8 Axis as a Novel Module for Targeted Intervention
Source: Adv Sci (Weinh). 2024 Nov 28;12(3):2407572. doi: 10.1002/advs.202407572 (PMC11744578; doi:10.1002/advs.202407572)
Supplement: Supplementary file 1 — Supporting Information [file ADVS-12-2407572-s001.docx]

**SUPPLEMENTARY MATERIAL**

**Supplementary Figure: 3**

**Supplementary Tables: 7**

**Figure S1. Composition of hepatic non-parenchymal cells from different donors.** **a**, Representative gating strategies for human liver non-parenchymal cells (NPCs). **b**, Absolute number of CD45^+^ liver immune cells per vial. Note that immune cells can hardly be passaged. **c**, Relative proportions of the main immune cell subpopulations. **d**, Relative proportions of monocyte/macrophage polarization phenotypes. **e**, Relative proportions of CD4/CD8^+^ T cell subsets among MAIT and non-MAIT cells and representative flow cytometry staining for CD4 and CD8. **f**, Relative proportions of NKG2A/KIR^+^ cell subsets among CD16^+/-^ NK cells and representative staining for KIR and NKG2A. DN = double negative; DP = double positive; MAIT cells = mucosal-associated invariant T cells; NK cells = natural killer cells; PBMC = peripheral blood mononuclear cells; SP = single positive.

**Figure S2. MASH liver spheroids show that elafibranor is anti-steatotic and anti-inflammatory without reducing fibrosis.** **a**, Representative images of MASH spheroids and spheroids treated with elafibranor for 7 days. Note that Elafibranor treatment significantly reduced steatosis. Small grey numbers indicate the number of analyzed spheroids. **** indicates P<0.0001 in heteroscedastic two-tailed t-test. **b**, Elafibranor treatment does not reduce the secretion of pro-collagen I. Error bars indicate SEM. **c**, Principal component analysis (PCA) of differential gene expression patterns based on RNA-Seq analyses of control spheroids, MASH spheroids and MASH spheroids treated with elafibranor. Results from three different donors are shown. **d**, Reactome pathway analysis. The dot sizes represent the number of differentially abundant transcripts within the pathway. **e**, Gene set enrichment analysis (GSEA) of lipid catabolism shows a borderline significant (false discovery rate [FDR] = 0.06) association with elafibranor treatment. NES = normalized enrichment score.

**Figure S3. Replication of chemogenomic hits. a**, Effects of the anti-steatotic hits PQCA (CHRM1 agonist), selumetinib (allosteric MEK1/2 inhibitor) and MK-2206 (allosteric AKT inhibitor). n=7-8. Asterisks refer to heteroscedastic two-tailed t-tests between PQCA and its inactive analogue (MSD-M1PAM-NC). ##, ###, #### indicate *p*<0.01, *p*<0.001 and *p*<0.0001 compared to untreated MASH cultures, respectively. **b**, Effects of the anti-fibrotic hits PQCA (CHRM1 agonist), TP-008 (TGFBR1 inhibitor), PF-05105679 (TRPM8 antagonist) and MK-2206 (allosteric AKT inhibitor). n=8-47. Data is shown as percentage of untreated MASH cultures (red dashed line). *, **, ***, **** indicate *p*<0.05, *p*<0.01, *p*<0.001 and *p*<0.0001, respectively. Error bars indicate SEM.

**Table S1. Donor characteristics.**

**Table S2. Overview of the tested chemogenomic probes and their targets.**

**Table S3. Results of Reactome pathway analyses at the transcriptomic level.**

**Table S4. Activity profiles of 500 transcription factors based on enhancer motif inference.**

**Table S5. Results of Reactome pathway analyses comparing MASH and control cultures at the proteomic level.**

**Table S6. Overview of all analyzed lipid species and their alteration in MASH cultures.** FC = fold change. AcCa = acetylcarnitine; Cer = ceramides; DAG = diacylglycerols; FA = fatty acids; LPC = lysophosphatidylcholines; PC = phosphatidylcholines; PE = phosphatidylethanolamine; PI = phosphatidylinositol; PS = phosphatidylserine; SM = sphingomyelin; TAG = triacylglycerols.

**Table S7. Hepatotoxicity counter-screen of chemical probes.**
