# Supplementary material for: Chemogenomic Screening in a Patient‐Derived 3D Fatty Liver Disease Model Reveals the CHRM1‐TRPM8 Axis as a Novel Module for Targeted Intervention
Source: Adv Sci (Weinh). 2024 Nov 28;12(3):2407572. doi: 10.1002/advs.202407572 (PMC11744578; doi:10.1002/advs.202407572)
Supplement: Supplementary file 2 — Supporting Figure and Table [file ADVS-12-2407572-s002.zip › Supplementary table 1_revision.docx]

**Table S1: Medical and demographic information of liver cell donors.**

| **Donor** | **Sex** | **Ethnicity** | **Age** | **BMI** | **Cause of death** |
| --- | --- | --- | --- | --- | --- |
| 1 | M | Caucasian | 63 | N/A | N/A |
| 2 | F | Caucasian | 39 | 35.1 | Anoxia |
| 2 | F | African American | 27 | 28.2 | Anoxia |
| 3 | F | Hispanic | 30 | 30.8 | Head trauma |
| 4 | M | Hispanic | 25 | 32.2 | Head trauma |
| 5 | F | Hispanic | 45 | 32.0 | Anoxia |
| 6 | N/A | N/A | N/A | N/A | N/A |
