# Supplementary figures and images for: Chemogenomic Screening in a Patient‐Derived 3D Fatty Liver Disease Model Reveals the CHRM1‐TRPM8 Axis as a Novel Module for Targeted Intervention
Source: Adv Sci (Weinh). 2024 Nov 28;12(3):2407572. doi: 10.1002/advs.202407572 (PMC11744578; doi:10.1002/advs.202407572)

# Figure S1

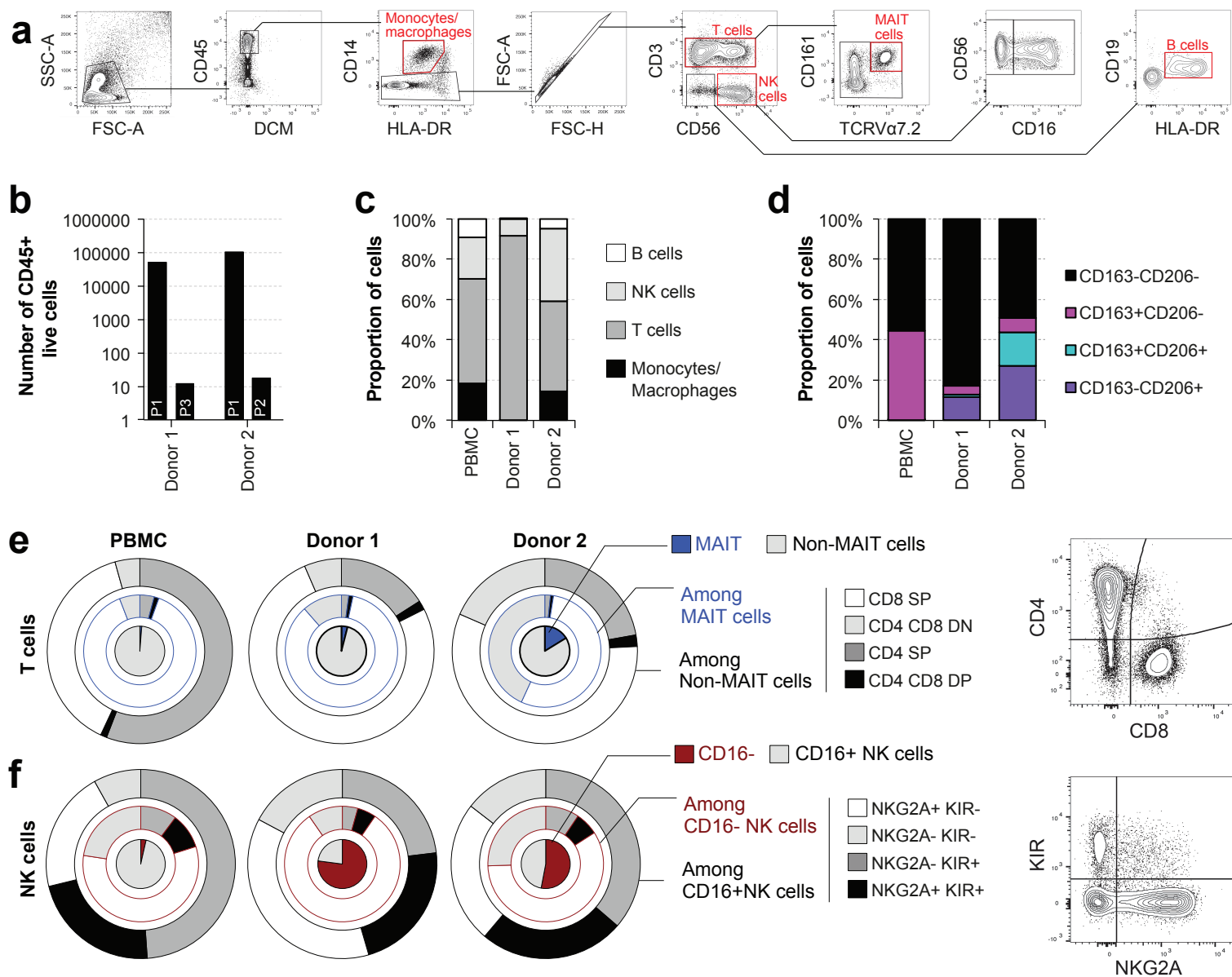

Supplement: Supplementary file 2 — Supporting Figure and Table [file ADVS-12-2407572-s002.zip › Supplementary Figure 1_revision.pdf]

# Figure S2

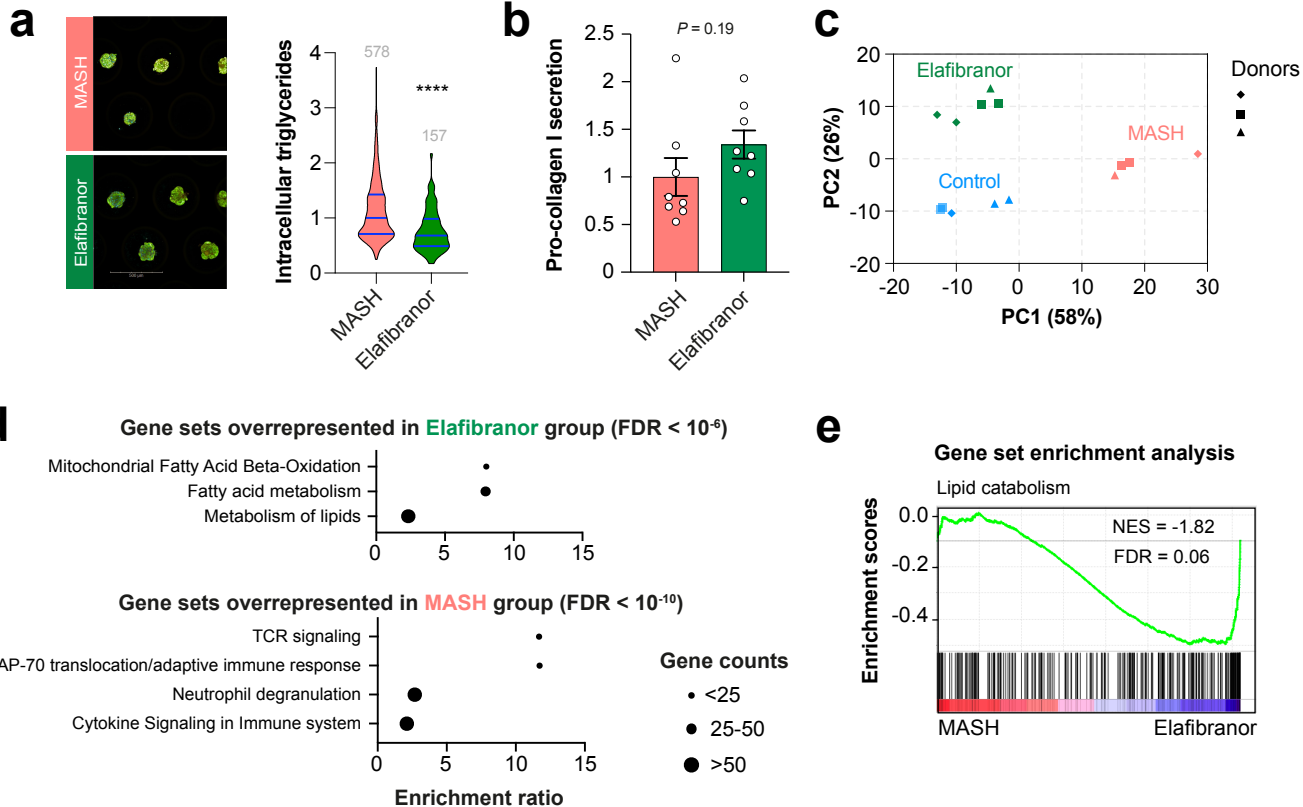

Supplement: Supplementary file 2 — Supporting Figure and Table [file ADVS-12-2407572-s002.zip › Supplementary Figure 2_revision.pdf]

# Figure S3

**a**

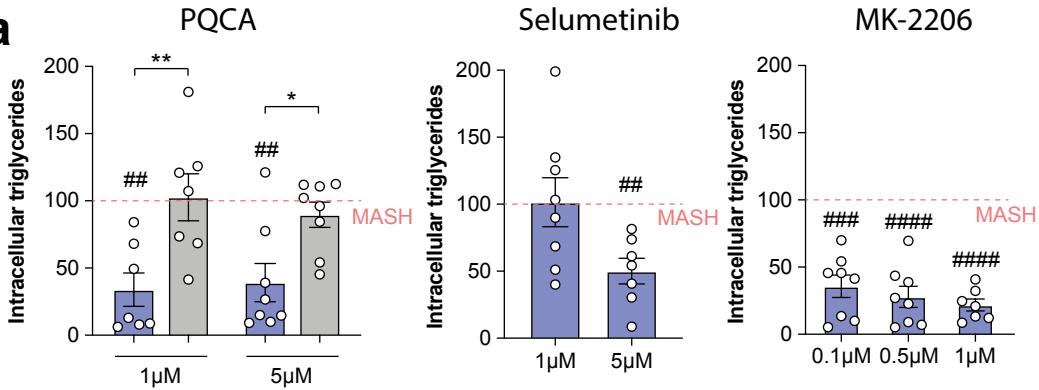

**b**

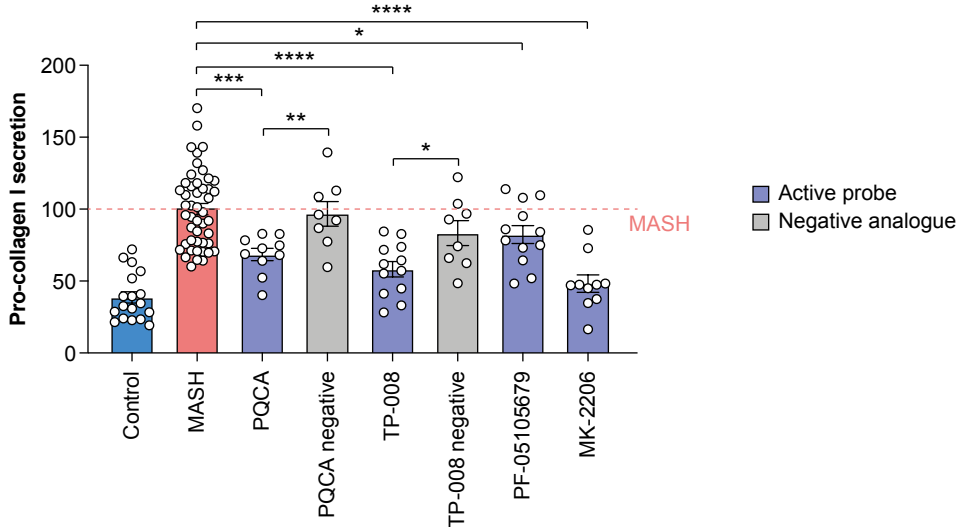

Supplement: Supplementary file 2 — Supporting Figure and Table [file ADVS-12-2407572-s002.zip › Supplementary Figure 3_revision.pdf]
